# Beyond feedback: 11 tips for coaching writing

**DOI:** 10.1007/s40037-020-00630-z

**Published:** 2020-11-24

**Authors:** Lorelei Lingard, Chris Watling

**Affiliations:** Centre for Education Research & Innovation, Schulich School of Medicine & Dentistry, Western University, London, Canada

In the Writer’s Craft section we offer simple tips to improve your writing in one of three areas: Energy, Clarity and Persuasiveness. Each entry focuses on a key writing feature or strategy, illustrates how it commonly goes wrong, teaches the grammatical underpinnings necessary to understand it and offers suggestions to wield it effectively. We encourage readers to share comments on or suggestions for this section on Twitter, using the hashtag: #how’syourwriting?

We all give feedback on others’ writing. Usually we do so as co-authors, supervisors, or pre-submission friendly readers. Such feedback almost always makes writing better, particularly if we apply strategies to maximize its effectiveness [1]. Sometimes, however, we’re doing more than providing feedback on a draft. Sometimes we’re coaching other researchers as writers.

‘Coaching’ is not a word that health researchers commonly use in relation to their writing, so you might need to stop and think about this for a minute. Do you have someone who reads most if not all of your writing, who knows your writing processes as well as your products, who has helped you evolve as a writer? Or perhaps you *are* that person for a peer, junior colleague or graduate student: is there someone whose ‘voice’ you have watched develop over many manuscripts? Someone whose writing style you recognize well enough to know when they’re trying something new or struggling with a longstanding challenge?

Chances are that you will have said ‘Yes!’ (or ‘Hmmm, maybe …’) to one or all of these questions. Now ask yourself, is that coaching relationship purposeful or accidental? Accidental doesn’t necessarily mean ineffective, but we believe that writing coaching is empowered when it is conscious, explicit and planned. Emerging evidence suggests that writing coaching can improve health researchers’ productivity and their satisfaction [2]. This Writer’s Craft instalment offers 11 tips to foster productive coaching interactions.

1. **Get to know each other as writers**. Share a piece of your own writing that you’re proud of. Discuss a paper you found particularly effective. Talk about your writing aspirations—such as your goal to publish original research in the BMJ—and your struggles—such as those paragraphs that take a whole day to write only to be deleted the next morning. Such sharing demystifies writing for novices. It also explicitly rejects the culture of secrecy and competitiveness around academic writing that can make it inhospitable to newcomers [3].
2. **Create a safe space**. Coaches need to provide a safe place for writers to develop, to confront their insecurities, and to become confident enough to improvise within the rules and find their own voices. Give writers permission to submit ‘shitty first drafts’ [4] when the focus is more on thinking. Manage expectations by acknowledging that there will be many rounds of drafting and feedback before a final product emerges. Agree on the focus for a particular round of writing review—story, structure, or style [5]—and overlook the other aspects for now. Share reviewers’ comments and discuss how to interpret and respond to them.
3. **Acknowledge vulnerability**. Coaches can feel they are being enthusiastic and responsive while writers just feel naked and exposed. Coaches should try to notice when writers seem particularly vulnerable. Get into the habit of asking, ‘How challenging does this feel for you?’ Let the writer choose the focus sometimes: ‘What are you working on in this piece of writing?’ And, if writing in English as an additional language is a factor, talk explicitly about this aspect of the challenge. Ask if the writer thinks their voice ‘sounds different’ in English than in their first language, and discuss how that affects their developing sense of themselves as a writer.
4. **Clarify roles and revisit them regularly**. The roles of coach and writer are not static: they may need to be refreshed with reference to the specific writing task, the writer’s stage of development, and the trajectory of the relationship. Make roles explicit and revisit them as the context evolves. If you coach multiple writers simultaneously, tailor your role and expectations to each unique situation. And don’t assume ‘once a coach, always a coach’. Outside the graduate supervisor role with its clear start and end, the coaching role can be a bit murky. Coaches might need to ask: ‘Am I *still* coaching this writer?’; ‘Am I coaching this writer *in this situation*?’
5. **Work on the thinking**. ‘Writing is thinking … that’s why it’s so hard’ [6]. Writing isn’t the only product you’re working on together. Particularly when you are giving multiple rounds of feedback on work-in-development, you are coaching the thinking as well as the writing. Try to be explicit about which is in the foreground at any point in time: for instance, ‘I think this is a snarl in the thinking/logic more than a problem with the writing. Let’s play with that a bit more.’ Remember, though, critiquing how someone thinks can be even more threatening than critiquing how they write. Critique should be characterized by generosity and grace.
6. **Talk it out**. Speaking is a powerful adjuvant to writing. When you find yourselves at a conceptual crossroads (or, even worse, in a conceptual swamp), put the draft aside and chat about the story. Brainstorm, play with the ideas, summarize back to each other what you think you understand. It can be easier to relinquish past ideas and structures when you haven’t committed them to the page. Audio-record these sessions if it’s helpful for the writer, but the goal is not phrasing you can cut and paste into a draft: it’s coherence of ideas.
7. **Address attitudes**. Writing success happens in the head before it translates to the page. Good writers talk themselves into writing, whether they feel like it or not, whether they know what to write or not, whether they like or dislike what they see appearing on the screen. Writers have to *believe*. Therefore, coaches need to understand writers’ beliefs about writing. These beliefs tend to cluster around three issues that coaches should probe: readiness (‘It’s not time to write yet’), clarity (‘I need to get it clear in my head first’) and quality (‘Whatever I write is going to be awful’) [7].
8. **Consider process**. Good writing practice is the product of multiple conditions, many of which we never subject to critical scrutiny. Things like when and where we write can have powerful, and largely unnoticed, impacts on our process. Helen Sword’s treatment of the ‘behavioral habits’ of ‘time, space and ritual’ [8] is a good starting place for a discussion about when, where and how we write. Coaches can ask writers to photograph their writing space and analyze how its configuration shapes their writing. Changes to behavioral routines can be trialed: evening writers can try a morning routine. Or ‘binge writers’—those who wait till they have whole days available to write—can experiment with 60 min of ‘snack writing’ daily [9].
9. **Engage in reflection**. Every writer knows how to agonize about their writing. But reflecting productively can be more difficult. Coaching, with its ability to analyze, to question, to offer alternatives, can help writers learn to reflect more productively. By asking questions—Why do you feel that way about writing? How have you come to organize your writing projects this way? Why do you think reviewers reacted as they did? Why do you think it’s difficult for you to change this habit?—coaches help writers to develop a critical stance on their own process.
10. **Attend to identity**. Helping health researchers to develop a sense of themselves *as writers* is perhaps the most sophisticated aspect of writing coaching. Text work and identity work are entangled [10], so that when we shape the writing we are invariably shaping the writer. Writing scholars recognize that such shaping ‘is unnerving—at times excruciating’ [11, p. 194]. Coaches can attend to identity work by explicitly acknowledging that identity is multi-faceted and encouraging writers to experiment with different voices and genres. For instance, the health researcher who is an internist, a clinical educator and musician with a passion for arts and humanities should be encouraged to weave these into their writing in meaningful ways.

Finally, we offer this advice as a fundamental principle for coaching relationships:

11. **Remember, it’s ultimately about the writer**. Coaches offer advice, expertise, and support, but what writers do with those offerings is up to them. Coaches can become quite invested in the work, coming to see it as a reflection of their own identity. This is understandable but must be navigated carefully. If the paper starts sounding like the coach rather than the writer, that’s a red flag. If you find yourself in this situation (and trust us, you will, despite the best laid plans), step back and have a discussion about what has happened and why. Remember, a great coaching performance draws out another person’s unique character and skill, it doesn’t duplicate our own.

## Conclusion

The best coaches—in sports, in music—are judged not by their own performance, but by the performances of those whom they coach. If we can coach writing well, then we stand to make health researchers more productive and skilled, to give them confidence in their identity *as writers*, and to infuse their writing work with satisfaction and joy.
